# Development of an Experimental Ex Vivo Wound Model to Evaluate Antimicrobial Efficacy of Topical Formulations

**DOI:** 10.3390/ijms22095045

**Published:** 2021-05-10

**Authors:** Madelene Å Andersson, Lone Bruhn Madsen, Artur Schmidtchen, Manoj Puthia

**Affiliations:** 1 In2Cure AB, Medicon Village, SE-22381 Lund, Sweden; madeleine_a.andersson@med.lu.se; 2 Timeline Bioresearch, Medicon Village, SE-22363 Lund, Sweden; lbm@pipeline-biotech.dk; 3 Copenhagen Wound Healing Center, Bispebjerg Hospital, Department of Biomedical Sciences, University of Copenhagen, DK-2400 Copenhagen, Denmark; artur.schmidtchen@med.lu.se; 4 Dermatology, Skåne University Hospital, SE-22185 Lund, Sweden; 5 Division of Dermatology and Venereology, Department of Clinical Sciences, Lund University, SE-22184 Lund, Sweden

**Keywords:** ex vivo, wound model, antimicrobial, biofilm, burn, antibiotics, wound healing

## Abstract

Wound infections are considered a major cause for wound-associated morbidity. There is a high demand for alternative, robust, and affordable methods that can provide relatable and reproducible results when testing topical treatments, both in research and in the pharmaceutical industry. Here we present an ex vivo wound infection model using porcine skin and a burn wounding method, allowing for the efficacy evaluation of topical antimicrobial formulations. Utilizing this model, we demonstrate the potential of topical treatments after infecting the wounds with clinically significant bacteria, *P. aeruginosa* and *S. aureus*. We show that the method is compatible with several analytical tools used to analyze infection and antimicrobial effects. Both bacterial strains successfully infected the wound surface, as well as deeper regions of the tissue. Quantification of viable bacteria on the wound surface and in the tissue, longitudinal measurements of bioluminescence, fluorescence microscopy, and scanning electron microscopy were used to confirm the effects of antibacterial treatments. Furthermore, we show that biofilms are formed on the wound surface, indicating that the demonstrated method mirrors typical in vivo infections.

## 1. Introduction

Over the last 25 years, more than one thousand in vitro and in vivo wound models have been documented and proven useful for the study of various aspects of wounding [1,2,3,4,5,6,7,8]. A literature review found that, since 1993, 74% of wound models were in vivo models, followed by in vitro wound assays representing 23% of the documented methods [1]. In vitro methods are simple, well controlled, standardized, high throughput, affordable, and involve fewer ethical considerations compared to working with laboratory animals or humans [9]. However, given the complexity of the interplay between the host immune system, tissue heterogeneity, wound bed components, and the infectious bacteria, in vitro systems alone are not optimal to mimic mechanisms involved in wounds [4,10]. For this reason, animal models have become the gold standard tool for experimental studies of various aspects of external wounding, commonly using mouse, rat, pig, rabbit, dog, sheep, and goat models [3]. Even though in vivo methods are most commonly used, difficulties in studying wound infections in animals makes their results difficult to interpret [11]. Factors such as irreproducible wounding and infection, difficulties in sampling, inability to collect longitudinal biopsies from living animals, and difficulties in application of topical treatment formulations (ointment or gels) and wound dressing (especially in small animals) make in vivo wound studies complicated to perform. In addition, most in vivo wound models go through stringent ethical scrutiny and are expensive to accomplish. Therefore, in the wound research and pharmaceutical development fields, there is a high demand for alternative, robust, high throughput, and affordable models that could be used to evaluate the efficacy of topical treatments.

Less than 3% of the reviewed wound models were ex vivo models [1]. These models are typically based on tissues and organs collected from living organisms, and experiments are then conducted under artificial conditions. Even though ex vivo models are used infrequently, they can be advantageous because they allow for more controlled experimental conditions and are covered by fewer ethical concerns compared to in vivo studies, while representing more natural conditions compared to in vitro methods [2]. There is a high degree of phenotypic variation in skin characteristics between animals that must be taken into account when studying skin wounding. An accurate wound model should therefore utilize animal species with similar skin characteristics to human skin [5]. The Wound Healing Society recommends the porcine skin model for pre-clinical studies of wounds, as it closely resembles human skin [12], and as pigs share a higher similarity to humans in their immune systems compared to rats and mice, thus providing comparable interactions between infection and immune system components [5,6,13].

There is an unmet need for simple ex vivo model-based screening methods that can be used to study wound infection mechanisms and to evaluate efficacies of antibacterial treatments in pharmaceutical development. The model presented herein has the potential to meet these needs. This ex vivo method utilizes previously frozen porcine skin and a burn wounding procedure to create reproducible partial thickness wounds on which we demonstrate a successful bacterial infection using either *P. aeruginosa* or *S. aureus,* the two most common bacteria found in infected wounds [14,15,16]. Furthermore, we show migration of the bacterial cells into the deeper regions of the tissue, mimicking an in vivo infection that is applicable to different kinds of wounds. Using two common antimicrobial treatments, we attempt to treat the infected wounds. After two hours of treatment of the pre-infected skin, we show reduced bacteria, both topically and intra-tissue. Using in vivo imaging system (IVIS), we also demonstrate longitudinal monitoring of the infection on the wound surface.

## 2. Results

### 2.1. Characteristics of Burn-Induced Ex Vivo Wounds

**Figure 1:**
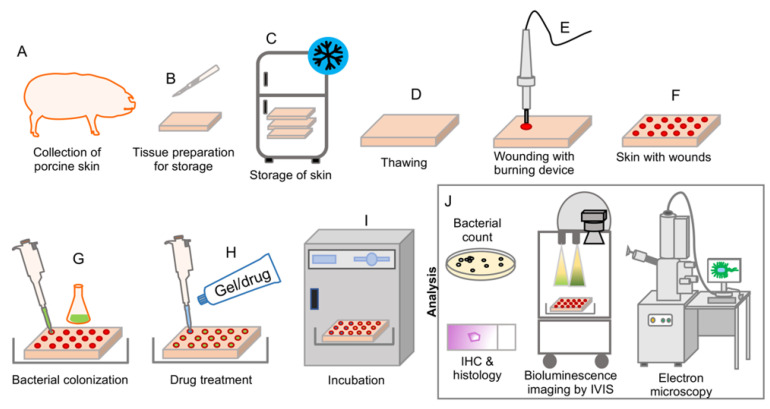
Graphic illustration demonstrating the experimental set-up and workflow of the ex vivo wound model. (**A**) Skin was shaved, cleaned, and surgically collected from the backs of euthanized Göttingen minipigs. (**B**,**C**) Prior to storage, the skin samples were cut into suitable sizes and packaged into aluminum foil and plastic bags, after which the packages were stored at −20 °C until use. (**D**) Skins were defrosted and thawed at room temperature for two hours and excess fat was removed using a scalpel. (**E**,**F**) A soldering iron had been preheated for 10 min to obtain a temperature of ~368 °C. The tool tip was placed against the skin and held with a uniform pressure for 15 s to create burn wounds. (**G**) Infection was established by adding 30 µL of 10^8^ CFU/mL of *S. aureus* or *P. aeruginosa* to the wound surface. For experiments with bioluminescent measuring, bioluminescent strains were used at this step. After addition of the bacterial solution, the skin was incubated for 2 h at 37 °C to allow the infection to become established. (**H**,**I**) One hundred µL of antibacterial treatments were added to each wound, and the skins were placed back into the incubator for an additional 2 h. (**J**) After incubation with the applied treatment, samples were prepared for further analysis by several different methods. Methods used included viable bacterial count assay, histology and immunohistochemistry, and SEM. Longitudinal bioluminescence imaging using IVIS was conducted as repeated measurements during the treatment period.

**Figure 2:**
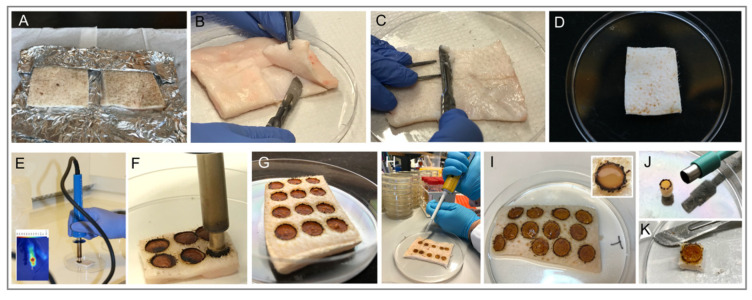
Photographs of the ex vivo wound model during the various procedural steps. (**A**) Frozen pig skin grafts left to defrost on the aluminum foil packaging for 2 h before continued handling. (**B**) Excess fat being separated and removed from the skin using a scalpel. (**C**,**D**) Residual fat remaining on the skin being removed by scraping with a scalpel, creating a homogenous tissue thickness and preventing potential bulging regions on the skin. (**E**,**F**,**G**) The soldering iron with a Ø 8 mm tip being placed against the skin for 15 s, creating uniformly sized wounds. (**H**) Bacterial solution being added to each of the wounds using a pipette. (**I**) Infected wounds immediately after application of antibacterial treatments. (**J**) Wound tissue collected using biopsy punch. (**K**) A piece of wound tissue cut out from the skin using a scalpel at the end of the experiment prior to preparation for various downstream analyses.

Under strict aseptic conditions, skins from Göttingen minipigs were collected and stored in a −20 °C freezer (Figure 1). Frozen skin was defrosted at room temperature and prepared for the wounding. An electric soldering iron was used to produce uniform burn wounds on the defrosted skin (Figure 1 and Figure 2). Ex-vivo wounds were infected with bacteria, and treatments were applied topically (Figure 1 and Figure 2). The complete method for the skin collection, preservation, burn wounding, infection, and treatment is described in the Materials and Methods section.

**Figure 3:**
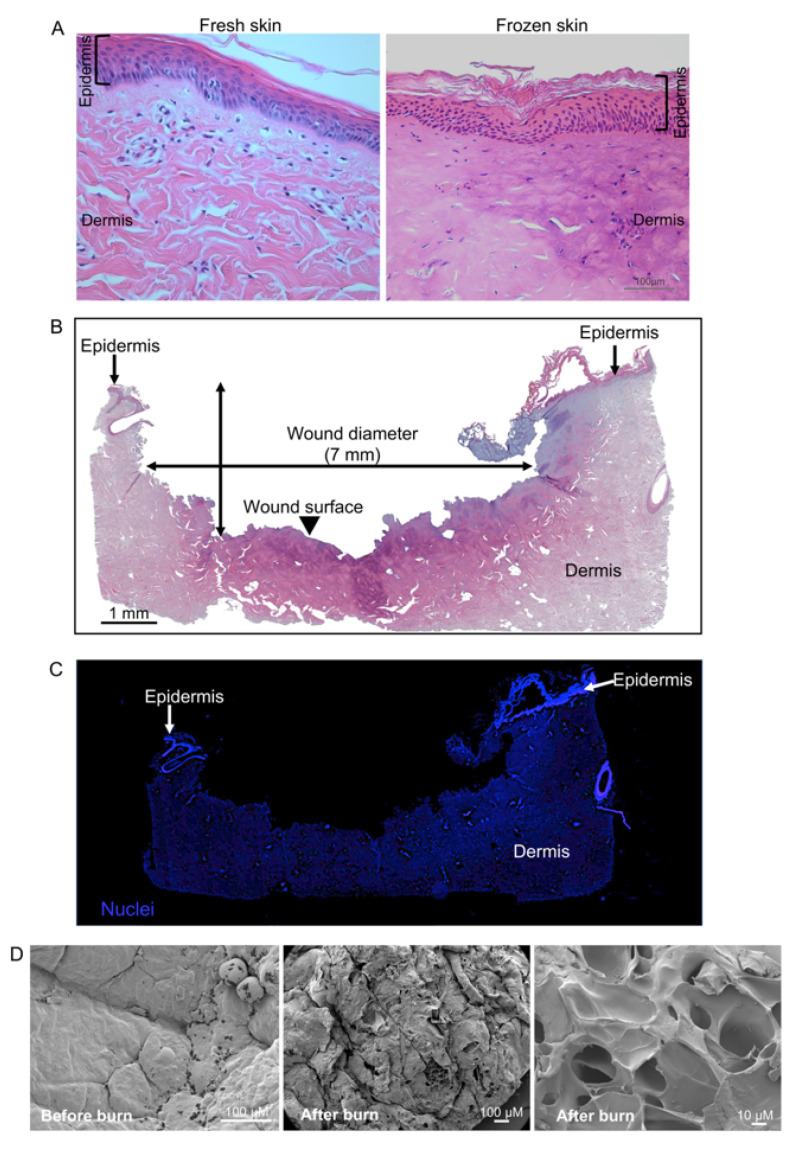
Histology and characteristics of burn-induced ex vivo wounds. (**A**) H&E stained sections showing histology comparing freshly excised and freeze-thawed porcine skin tissue. (**B**) Representative images showing panoramic composition of ex vivo wound tissue. Stitched image of H&E-stained wound tissue sections (containing images taken with a 5× objective) from an uninfected wound. Thin arrows indicate the undamaged epidermis surrounding the wound, while the arrowhead indicates the disrupted epithelium and severe tissue damage on the wound surface. The extent of the wound is indicated by the wound diameter. (**C**) Wounded tissue from the same sample stained with the general DNA stain, DAPI. The partial-thickness wound was imaged using fluorescence microscopy, showing the damaged tissue. (**D**) Scanning electron microscopy of surface of burn-induced ex vivo wound.

Histology of freshly excised and freeze-thawed porcine skin was compared to study if freeze-thawing had any adverse effect on the skin tissue (Figure 3A). Skin tissue after freeze-thawing did not show any apparent adverse effect and was comparable to the fresh porcine skin tissue. A well-defined epidermis and dermis were noticed, along with normal nuclei. No other freeze-thawing-related tissue artifacts were noticed. To demonstrate the characteristics of the wounds inflicted by burn method, H&E staining was used to visualize the wound architecture (Figure 3B). The image shown is representative for nine analyzed wounds, which shows a clear disruption to the epidermis and damage to the underlying layer of dermis, similar to what is seen in different types of partial thickness wounds [17,18,19]. A complete loss of epidermis and abnormal dermis architecture is noticed. Analysis of H&E stained sections of the wound tissue showed wound diameter and depth to be approximately 7 mm and 3 mm respectively (Figure 3B). Furthermore, nuclear staining was used to compliment histological observations (Figure 3C). A normal nuclei distribution was observed throughout the tissue section. SEM of wounded skin exhibited distinct differences in appearance and structure compared to the skin structure before burn wounding (Figure 3D). Unwounded skin showed a smooth surface, whereas wounded skin showed a rough surface with disruptions.

### 2.2. Effects of Antibacterial Treatment on Simulated S. aureus and P. aeruginosa Wound Infections

To demonstrate the possibility for this ex vivo method as a model for wound infection intervention studies, we infected the wounds with clinically important pathogens, either the Gram-positive *S. aureus* or the Gram-negative *P. aeruginosa*. *S. aureus* and *P. aeruginosa* are the most common pathogens found in infected wounds [14,15,16]. The use of *S. aureus* was also motivated by the fact that it is the most common pathogen causing wound infections post-surgery [20,21]. Moreover, it is also very common in non-healing wounds [22]. In context of burns, *P. aeruginosa* and *S. aureus* [20,21] are the most common cause of burn wound infections [15]. For the treatment of the wound infection, we aimed to use a topical gel (Prontosan) and a water-soluble antibiotic (Levofloxacin). Prontosan wound gel is a current standard benchmark in wound care and contains polyhexamethylene biguanide (0.1%) and undecylenamidopropyl betaine (0.1%). Prontosan gel is reported to reduce and prevent gram-negative and gram-positive wound infections and biofilm formation [23]. Levofloxacin is a clinically used antibiotic with a broad antimicrobial spectrum [24] and excellent soft tissue penetration properties [25,26].

**Figure 4:**
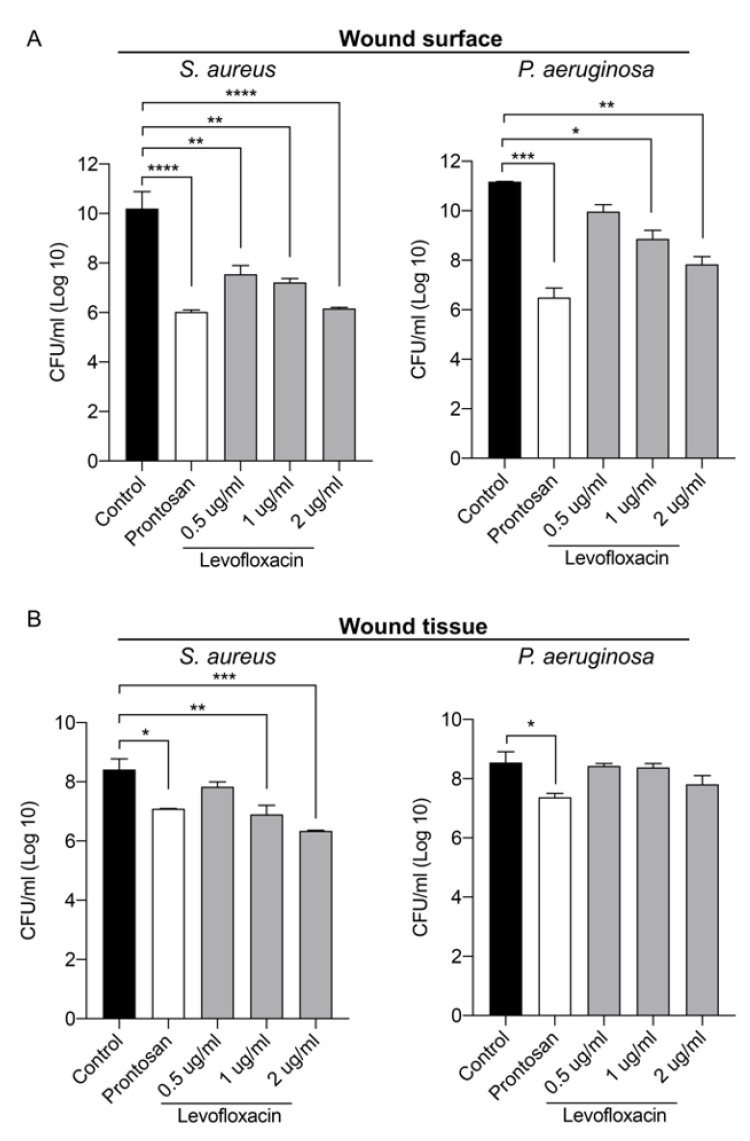
Evaluation of topical antimicrobial treatments in an infected ex vivo wound model. Infection was induced with either *S. aureus* or *P. aeruginosa* by adding 30 µL of a 10^8^ CFU/mL bacterial solution. Infection was allowed to establish for 2 h prior to addition of 100 µL of treatment (Prontosan or Levofloxacin at the indicated concentrations) to each wound. Tissues were then incubated for an additional 2 h prior to sampling. (**A**) Bar chart showing bacterial count from the wound surface. The wound surface was washed twice with PBS, and the number of colony forming units (CFU) in the wash solution was quantified. (**B**) Bar chart showing bacterial count from the wound tissue. Tissue samples were collected and homogenized and the number of colony-forming units in the tissue homogenate were quantified. Values were log_10_ transformed and are represented as the mean ± SEM (*n* = 3). *p* values were determined using a one-way ANOVA with Tukey post hoc test. Significant results are denoted as * *p* < 0.05, ** *p* < 0.01, *** *p* < 0.001, and **** *p* < 0.0001.

Two hours after the infection, Prontosan or Levofloxacin were applied topically on the wounds and viable bacterial counts were determined both on the wound surface and in the tissue (Figure 4). Both Prontosan and Levofloxacin significantly reduced the viable bacterial load on the wound surface for bacterial species compared to untreated controls (Figure 4A). Prontosan treatment reduced both *S. aureus* and *P. aeruginosa* in the wound tissue, while Levofloxacin reduced only *S. aureus* in the wound tissue (Figure 4B). Levofloxacin treatment showed a dose-dependent response, demonstrating the sensitivity of the method.

**Figure 5:**
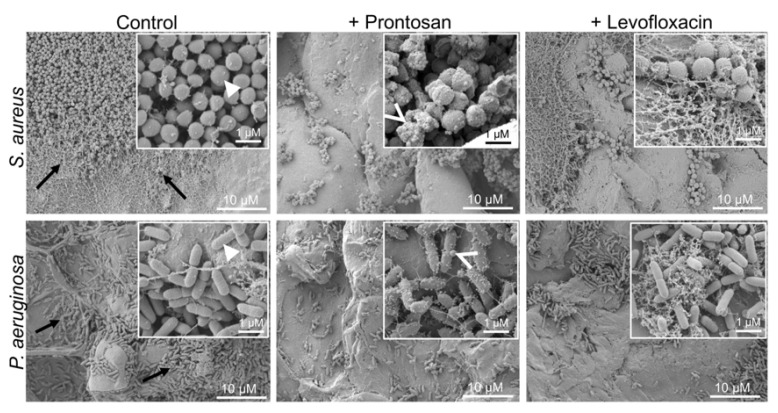
Scanning electron microscopy of surface of infected ex vivo wounds. Wounds were infected for 2 h with either *S. aureus* or *P. aeruginosa*. Infected wounds were then treated topically with Prontosan or 2 µg/mL Levofloxacin for 2 h. Biofilm formation and microcolonies are observed in the control wounds (arrow). Bacteria in control wound show smooth cell wall surface (arrowhead), and small perturbations (open arrow) were observed on bacterial surfaces in the Pronstosan group.

Infected and treated wounds were further visualized using SEM. Control wounds infected with *S. aureus* or *P. aeruginosa*, without antimicrobial treatment, had abundant bacterial colonies on the surface after 4 h (Figure 5). Both antibacterial treatments visibly reduced the number of bacterial cells on the wound surface, consistent with the reduced viable bacterial counts found in these wounds. Bacterial clumping and debris were observed in the Prontosan-treated wounds. Bacteria in control wounds showed smooth cell wall surfaces, whereas small perturbations were observed on bacterial surfaces in the Prontosan group. The SEM images also show a primary formation of bacterial biofilms and microcolonies on the control wound surface, which appears to be disrupted after treatment, particularly with Prontosan.

### 2.3. Longitudinal Evaluation of Antibacterial Treatment Using Bioluminescence Imaging

**Figure 6:**
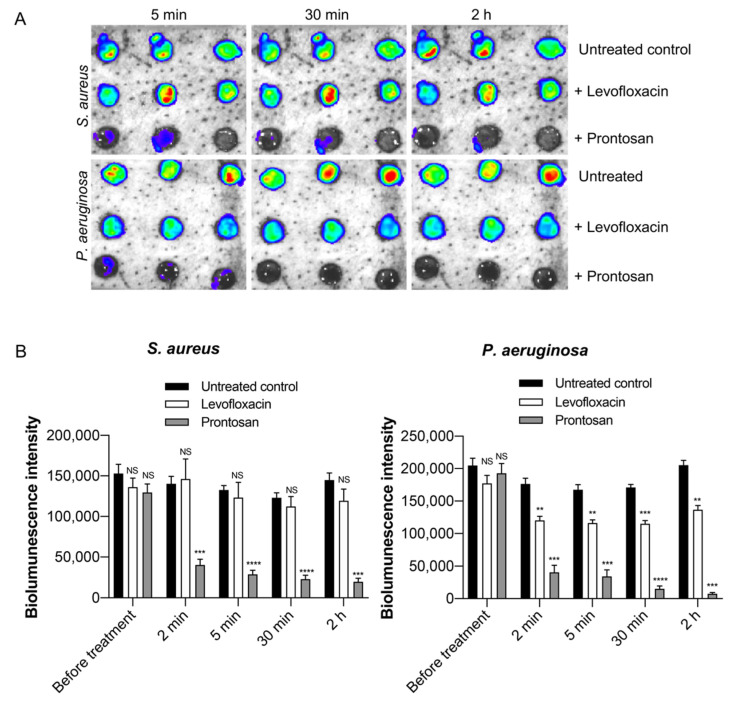
Longitudinal infection imaging of ex vivo wounds. (**A**) Wounds were infected with bioluminescent strains of *S. aureus* or *P. aeruginosa*, incubated for two hours, and treated with the antimicrobial agents Prontosan or 2 µg/mL Levofloxacin. Bacterial bioluminescence intensity was non-invasively analyzed using the IVIS bioimaging system. Representative images show bacterial luminescence. (**B**) The bar chart shows measured bioluminescence intensity emitted by the bacteria. Data are represented as the mean ± SEM (*n* = 4). *P* values were determined using a two-way ANOVA with Tukey test. Each group is compared to their respective untreated controls. Significant results are denoted as ** *p* < 0.01, *** *p* < 0.001, and **** *p* < 0.0001. NS, not significant.

To image the infections longitudinally, wounds were infected with bioluminescent *S. aureus* or *P. aeruginosa* bacteria and monitored over time using IVIS imaging (Figure 6A,B). Live imaging of infection allows for valuable kinetic studies. Measurements taken prior to addition of treatments showed that similar levels of infection were present in all wounds. After treating the wounds with antibacterial agents, repeated imaging allowed us to follow changes in the bacterial infection levels as a result of treatment. Control wounds showed stable bacterial bioluminescent intensity over the duration of the experiment, demonstrating a presence of healthy bacterial cells without additional supplementation of artificial nutrition. Prontosan-treated wounds showed a clear reduction in bioluminescence for both *S. aureus* and *P. aeruginosa*, indicated by a decrease in bioluminescent intensity over time. Levofloxacin treatment of wounds infected with only *P. aeruginosa* showed a significant decrease in bioluminescence.

### 2.4. Visualization of Bacterial Infection in the Wound Tissue

**Figure 7:**
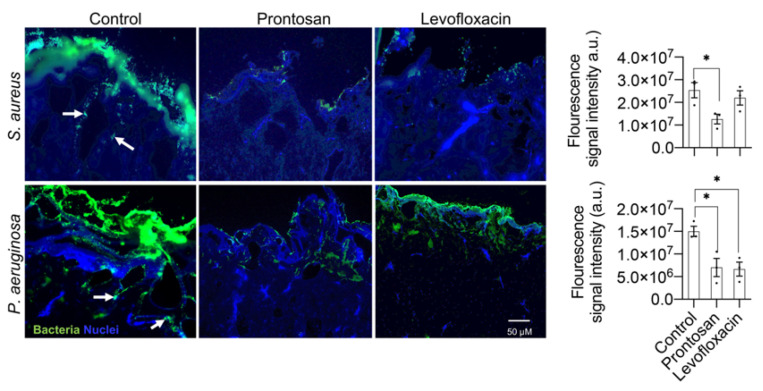
Immunofluorescence visualization of *S. aureus* and *P. aeruginosa* in infected wounds. Wounds were infected with *S. aureus* or *P. aeruginosa*, incubated for two hours, and treated with the antimicrobial agents Prontosan or 2 µg/mL Levofloxacin. After 2 h of treatment, the wound tissue was embedded and frozen in OCT for cryosectioning. Cryosections were then stained with the rabbit-anti-staphylococcus aureus IgG or rabbit-anti-pseudomonas IgG primary antibodies. An alexa-fluor-568-labeled goat-anti-rabbit IgG was used as secondary antibody. To visualize nuclei (blue), sections were counterstained with DAPI. Representative images are shown (*n* = 3). Arrow shows bacterial invasion in the tissue. Fluorescence intensity was measured as integrated density of the fluorescence signal and represented as arbitrary units (a.u.). Data are presented as mean ± SEM (*n* = 3). Significant results are denoted as * *p* < 0.05.

To study bacterial tissue invasion and the effect of treatments, immunofluorescence staining of *P. aeruginosa-* and *S. aureus-*infected wound tissue was performed using specific antibodies. Control untreated wounds showed a thick layer of bacteria present on the wound surface, which was observed for both bacterial strains used (Figure 7). Furthermore, bacterial cells were found to migrate into deeper regions of the tissue, with *P. aeruginosa* seemingly being more efficient in penetrating deeper in the tissue compared to *S. aureus*. In agreement with previous results, wounds treated with antimicrobial agents clearly demonstrated a decrease in fluorescence intensity compare to controls suggesting a reduction in infection both on the surface of the wound and within the tissue (Figure 7).

## 3. Discussion

In this paper, we describe an ex vivo infection model that can act as a bridge between in vitro and in vivo models. In vivo models are certainly unavoidable in preclinical and clinical phases. Our proposed ex vivo model provides a relatively robust method for early screening of antimicrobial compounds and formulations. Using several analytical tools, we demonstrate a high reproducibility in both wounding and infection characteristics. Our model provides several valuable features that are not present in vitro, while providing more controllable conditions and fewer ethical concerns compared to animal studies. Porcine skin has a high similarity to human skin anatomy and physiology [5,6,12,13], and the model could be easily adapted for the use of ex vivo human skin to further increase its clinical relevance. The use of ex vivo skin provides wound bed components that are naturally occurring in skin wounds, without the addition of supplementary growth media, providing a distinct advantage over in vitro models. Compared to in vivo models, the amount of treatment applied is easily controlled, and the treatments are left undisturbed. Animal behaviors such as scratching are often observed after wound dressing and can adversely affect the study. Moreover, contamination of animal wounds with feces or urine is a constant risk in wound studies. Most ex vivo models require fresh tissue, which has to be utilized immediately. Most of the precious animal tissue is wasted if not used immediately. Our method uses frozen skin tissue that can be stored frozen for months and used as required.

We demonstrate the use of this ex vivo model to study bacterial wound infections using clinically relevant bacterial strains, *P. aeruginosa* and *S. aureus,* the two most common bacteria found in infected wounds [14,15,16]. These two species have also been found to dominate polymicrobial biofilms in chronic wounds [16]. Through bioluminescent monitoring of the infection in the wounds, we showed that bacteria were thriving without addition of extra nutrients, maintaining a steady bacterial load through the course of the experiment. Consistent with previous findings in engineered skin tissue [27] and tissues taken from human chronic wounds [28,29], *P. aeruginosa* demonstrated higher invasion into deeper regions of the tissue, compared to *S. aureus.* Furthermore, the two common antimicrobial treatments used in the present study reduced bacteria on both the wound surface and inside the tissue, albeit with varying degrees. Collectively, these data demonstrate the high potential for this ex vivo model to be used also as a tool to study the penetrating properties of both pathogens and drugs into soft tissues, especially small molecule therapeutics. This model can be used for the screening, development, and evaluation of therapeutics. The burn wounding approach gives this model flexibility to easily change wound size and depth. Moreover, this ex vivo model is optimal for the application of various forms of treatments, such as solutions, gels, ointments, and dressings, thus allowing for a wide range of application possibilities. The treatment application method (i.e., topical) is also mimicked, which may provide drug distribution profiles similar to the in vivo situation. It is noteworthy that frozen ex vivo skin models have successfully been used for drug penetration and absorption studies [30,31].

There is a clear relationship between wound infection and biofilm formation [32,33,34]. Over 60% of patients with chronic wounds were found to have biofilm present on the wound bed [33]. The wound bed of a burn injury is initially sterile [15,35]; however, blisters, loose skin, slough, and necrotic tissue provide a microenvironment that is favorable for bacteria to colonize [36,37]. Much effort has been expended in developing methods to study biofilm formation and antibiofilm treatments. The majority of biofilm models currently use in vitro methods [38], which do not reflect the complex microenvironment that is naturally occurring in the wound bed [39]. Using ex vivo porcine skin to host mature biofilms, Wilkinson et al. [40] depicted biofilms in SEM images as long strings or clouds of extracellular polymeric substances (EPS), as well as the formation of microcolonies. Further studies conducted both in vitro and in vivo describe these microcolonies and long, branched EPS strands that are attached to the cell surfaces in biofilm structures [41]. Analyzing the SEM images of the burn wounds, we found similar structures as described for biofilm formation in vivo and in vitro, in wounds infected with *S. aureus* and *P. aeruginosa* in our ex vivo porcine skin model, indicating that initial biofilm formation had taken place on the wounds. Considering that necrotic tissue and debris is the ideal substrate for biofilm formation, it is not surprising that the burned necrotic tissue on the surface of our wound model would support biofilm formation, which could prove highly valuable for the study of antimicrobial effects on biofilm formation. It is notable that other studies have also reported the successful growth of biofilm on frozen porcine skin [42]. Furthermore, it was shown that in-vitro-determined MIC of Levofloxacin for the bacteria used in this study was not sufficient to eradicate the bacteria in the wounds. Bacterial cells in the wounds could be protected by the biofilm, making our model more biologically relevant and able to be used to realistically study the efficacy of compounds.

Other ex vivo models of skin wounding have been described. Wounds created by using scalpels or biopsy punches to wound porcine skin were used to investigate treatments of soft tissue infection, such as antimicrobial peptides [43,44], disinfectants [45], various wound dressings [46], and silver bioactive glass [47]. These studies report a good correlation between results from ex vivo and in vivo models, indicating that ex vivo models are of high potential value. However, the wounding methods described in these studies are limited in their ability to produce reliable and reproducible wounds [48]. Wounding methods such as biopsy punches or scalpels may provide inconsistent results, as they produce irreproducible wound depths and wound surface. In contrast, we use a burning tool under controlled conditions, which produces reproducible wound size, depth, and surface, and consistent results. The burned necrotic tissue in the proposed wound model provides an ideal surface for microbes to grow and form biofilm. Existing ex vivo models do not mimic a burn wound surface with charred necrotic tissue similar to burn patients, and therefore our model could provide additional tools for studying burn-associated infections at early stages. Moreover, this model can also be used to study methods for the debridement of necrotic tissue.

Notably, there are limitations of ex vivo models that restrict the type of studies for which these methods can be applied. Most ex vivo models lack a full range of host responses, and experiments can be conducted for shorter time periods in comparison to in vivo models [2,49,50]. As the model presented here uses frozen porcine skin, there is no active immune system in the tissue, and therefore it cannot reflect the complex in vivo wound-healing process. Information from this model can be used to understand modulation of topical bacterial loads and drug penetration, but not for wound-healing outcomes or transcriptional events. We have tested this method only for shorter time periods, and longer incubation time resulted in tissue degradation. Experiments with longer time periods may require optimization of the thawing process and supplementation with culture mediums. Extracellular matrix is fairly preserved in ex vivo models [51], and it can provide valuable information in the proposed model. In a skin biopsy ex vivo model, we have previously shown that *P. aeruginosa* infection caused severe destruction of collagen fibers [52]. In the current work, we have not evaluated the impact of infection on collagen fibers, and this should be the aim of future studies.

In summary, we have developed an ex vivo wound infection model using porcine skin and a burn wounding method. This new ex vivo model can be used to evaluate the efficacy of various topical antimicrobial formulations.

## 4. Materials and Methods

### 4.1. Bacteria and Growth Conditions

Bacteria were cultured on Todd Hewitt (TH) broth (BBL^TM^, BD, Sparks, MD, USA) solidified by 15 g/L of BactoAgar (Saveen Werner AB, Limnhamn, Sweden) at 37 °C overnight, and then kept in 4 °C until cultivation. Single bacterial colonies were taken from the cultures and inoculated in 5 mL of TH broth at 37 °C on a shaking incubator overnight. The following day, the bacterial culture was refreshed in 5 mL TH-broth and grown to logarithmic phase at OD_600_ = 0.4 at 37 °C on a shaking incubator. After washing of the bacterial pellet in 10 mM Tris (pH 7.4), a bacterial solution with 1 × 10^9^ colony forming units (CFU)/mL was prepared using 10 mM Tris pH 7.4. The bacterial strains used in this study include *S. aureus* (ATCC 29213; American type culture collection, Manassas, VA, USA) and *P. aeruginosa* PAO1 (ATCC 27853, Manassas, VA, USA). The bioluminescent strains *P. aeruginosa* Xen41 (PerkinElmer, Waltham, MA, USA) and *S. aureus* SAP229 (provided by Dr. Roger Plaut, Division of Bacterial, Parasitic, and Allergenic Products, FDA, Bethesda, MD, USA) were also used in experiments studying bioluminescent imaging.

### 4.2. Chemicals

For the treatment of the wound infection, we used two common wound treatments, one topical gel and another water-soluble antibiotic, Prontosan^®^ Wound Gel (0.1% Betaine, 0.1% Polyhexanide; BBraun, Hessen, Germany) and Levofloxacin (Sigma Aldrich, Saint Louis, MO, USA). Three concentrations of Levofloxacin, 0.5, 1, and 2 µg/mL, were used. Levofloxacin doses were based on previously reported MIC values for the strains used in this study, MIC*_S. aureus_* = 0.5 µg/mL and MIC*_P. aeruginosa_* =2 µg/mL [53]. Levofloxacin was chosen based on its documented properties for soft tissue penetration [25].

### 4.3. Pig Skin Collection and Storage

Skins from female Göttingen minipigs weighing 14–20 kg were collected for the ex-vivo experiments. All procedures were performed following strict aseptic techniques by qualified veterinarians. Skin was collected from minipigs that were used in other wound-healing studies without any systemic infection or medications [54]. Minipigs were acclimatized for 1 week before collection of skin. Skins were collected within 2 h after termination (Figure 1A). Using a hair clipper, hair on the back was clipped and skin was cleaned with lukewarm water. The skin was then disinfected with 70% ethanol and dried with sterile gauze. Using a surgical scalpel, skin was excised in pieces of approximately 10 × 12 cm (Figure 1B). During collection, most subcutaneous fat was not excised along with skin. Skin pieces were packed in plastic zipper storage bags and kept on ice until all skin was collected. Immediately after collection, zipper storage bags were stored in −20 °C freezer until use (Figure 1C).

### 4.4. Wounding Method

On the day of wounding, the frozen porcine skin pieces were defrosted at room temperature for 2 h prior to handling (Figure 1D and Figure 2A). Using a scalpel, excess fat tissue was removed from under the skin (Figure 2B), and the bottom surface was smoothened to obtain a uniform skin thickness (Figure 2C). Prior to burn wounding, the skin was cleaned topically using 70% ethanol. An electric soldering iron (Biltema, Lund, Sweden) with a round copper tip (Ø 8 mm) was used to create burn wounds on the skin. The copper tip was disinfected with 70% ethanol and cleaned before use. The soldering iron was left to preheat for 10 min to reach its maximum temperature (~368 °C, measured using a Fluke TiS45 infrared camera (Fluke, Everett, WA, USA). The skin graft was placed in a petri dish (Figure 2D) in a fume hood. Holding soldering iron vertically, the hot copper tip was held against the surface of the skin for 15 s (Figure 1E and Figure 2E,F). No downward pressure was applied but the soldering iron was held firmly to prevent horizontal movements. To remove burned tissue from the tip, it was cleaned by manual wiping with clean tissue papers after each wounding. The tool was allowed to reheat for 2 min between each wound, allowing it to regain its maximum temperature. A gap of 5–6 mm was kept in between each wound (Figure 1F). Post-burning, the skin was kept in a petri dish (Figure 2G) containing phosphate buffered saline (PBS), covering one third of the skin thickness, to maintain moisture in the tissue during the procedure. The skin was allowed to cool at room temperature for 20 min before adding bacterial solutions.

### 4.5. Wound Infection

Thirty µL of 1 × 10^8^ CFU/mL of either *P. aeruginosa* or *S. aureus* was placed in the center of each wound, and then spread out using a pipette tip to cover the entire burn area (Figure 1G and Figure 2H). To prevent evaporation from the wounds, they were gently covered with a piece of parafilm. The petri dish containing the skin was then covered with its lid and incubated at 37 °C for 2 h.

### 4.6. Antibacterial Treatment

After infection was established, 100 µL of treatment solution (Prontosan gel or Levofloxacin solution) or PBS was added to the wounds (Figure 1H and Figure 2I). Again, a fresh piece of parafilm was placed on the skin to cover the wounds. The tissue was then again placed in the incubator (37 °C) for an additional 2 h (Figure 1I).

### 4.7. Sampling and Viable Count Assay

To quantify viable bacteria in the wound, samples were taken both topically from the wound surface and from the wound tissue to determine the antimicrobial effects of the treatments. For topical sampling, the wound was washed twice with 40 µL PBS buffer using a pipette, and washings were collected. Samples were then serially diluted and plated on TH agar (THA) plates. For tissue sampling, the wound was cut from the skin using a scalpel, and the sides were trimmed removing all non-wounded tissue (Figure 2J). Using scissors, the tissue was cut into smaller pieces before being placed in 2 mL screw-cap micro tube (Sarstedt, Helsingborg, Sweden), together with 500 µL of PBS and approximately 30 ceramic beads (Ø 1.4 mm) (Qiagen Gmbh, Hilden, Germany). Samples were homogenized using a Roche MagNA Lyser at 6000 rpm for four 30 s cycles, with cooling on ice for 1 min between cycles to prevent overheating. The sample was then serially diluted and plated on THA plates. Plates were incubated at 37 °C overnight, and the following day the resulting colonies were counted. All analytical methods used are depicted in Figure 1J.

### 4.8. Scanning Electron Microscopy (SEM)

For SEM imaging, 4.0 × 4.0 mm samples were cut from either undamaged tissue or from the center of the wounds, prior to or after treatments, using a scalpel. The samples were first washed once in 0.1M Sorensen’s phosphate buffer pH 7.4, then fixed in approximately 2 mL of “SEM fix” (0.1M Sorenson’s phosphate buffer pH 7.4, 2% formaldehyde and 2% glutaraldehyde) at room temperature for 20 min. After fixation, samples were washed once in 0.1M Sorenson’s buffer pH 7.4. Fixed tissue samples were then dehydrated in a graded series of ethanol (50%, 70%, 80%, 90% and twice in 100%). Dehydrated tissue samples were then critical point dried (Quorum, Laughton, United Kingdom) and mounted on 12.5 mm aluminum stubs. Finally, mounted samples were sputtered with 10 nm gold/palladium (80/20) in a Quorum Q150T ES turbo pumped sputter coater (Quorum, Laughton, UK). Samples were then scanned and imaged in a Jeol JSM-7800F FEG-SEM (Tokyo, Japan).

### 4.9. Histology

Wound samples containing both wound tissue and normal undamaged skin tissue were collected using a scalpel. Tissue samples were then directly placed in neutral buffered formalin (Sigma) and fixed overnight at 4 °C. After serial dehydration, the samples were embedded in paraffin blocks, sectioned using a microtome (Leica), and routine hematoxylin and eosin (H&E) staining was performed. Stained tissue sections were then imaged with bright field microscopy (Axioplan 2, Zeiss). Tissue sections from the same samples were also stained with 4′,6-diamidino-2-phenylindole (DAPI) and visualized and imaged using fluorescence microscopy (Axioplan 2, Carl-Zeiss, Oberkochen, Germany). For both H&E- and DAPI-stained sections, 174 images were taken of the sections, which were then stitched together to gain a panoramic view using Axioplan 2. Image J was used to determine the diameter of the wound.

### 4.10. Cryosectioning and Immunohistochemistry

Wound tissue samples including non-wounded tissue margins were excised and cryopreserved using Tissue-Tek^®^ optimum cutting temperature (OCT) compound. The tissue blocks were then kept at −80 °C until sectioning. Sections of 8 µm thickness were cut using a cryostat (Leica), and the sections were placed on Superfrost^®^ Plus microscope slides (Thermo Fisher Scientific, Waltham, MA, USA). After drying at room temperature, slides were placed in pre-cooled (−20 °C) acetone/methanol (1:1) solution for 20 min for fixation and permeabilization. Slides were washed with PBS (5 min × 3 times), then blocked using a 1% bovine serum albumin (BSA), and diluted in PBS for 60 min at room temperature. Slides were then incubated with the rabbit-anti-*Pseudomonas* IgG (ab68538, Cambridge, UK) or rabbit-anti-*Staphylococcus aureus* IgG (ab20920, Cambridge, UK) primary antibodies (1:200 in 1% BSA) at 4 °C overnight. The next day, slides were washed in PBS (5 min × 3 times) and incubated for 60 min with goat-anti-rabbit IgG (Alexa-fluor 568 conjugated, A-11036; Thermo Fisher Scientific, Fremont, CA, USA) secondary antibody (1:500 in 1% BSA). Slides were then washed in PBS (5 min × 3 times) and counterstained with DAPI solution (1 µg/mL in PBS) for 5 min, and then washed once in PBS. Slides were dried until excess liquid had evaporated and were then mounted using aqueous antifade fluorescence mounting media (Permafluor, Thermo Fisher Scientific, Fremont, CA, USA). Sections were visualized and imaged using a fluorescence microscope (Axioplan 2, Zeiss). Fluorescence intensity in the samples was measured using ImageJ [55]. Images were converted to gray scale and a ROI (region of intertest) was selected. The integrated density was measured for the ROI of each sample.

### 4.11. Imaging of Infection

In a separate experiment, bioluminescent strains of *P. aeruginosa* and *S. aureus* were used to infect the wounds. Imaging of infection was achieved using an in vivo imaging system (IVIS Spectrum, PerkinElmer, Waltham, MA, USA), prior to addition of treatments with PBS, Prontosan and 2 µg/mL Levofloxacin. IVIS imaging was performed under luminescent imaging mode with stage temperature set to 37 °C. Bacterial luminescence was again measured at 5 min, 30 min, and 2 h after treatments had been applied to the wounds. Bioluminescence from the wounds was detected and quantified using Living Image 4.0 software (PerkinElmer, Waltham, MA, USA).

### 4.12. Statistical Analysis

Groups were compared using one-way ANOVA (with Dunnett or Tukey post-hoc test) or two-way ANOVA (with Tukey post-hoc test) for multiple comparisons (Prism v9). Data are presented as mean ± standard error of the mean (SEM). *p* values below 0.05 were considered significant.

## References

[1] Parnell L.K.S., Volk S.W. (2019). The Evolution of Animal Models in Wound Healing Research: 1993–2017. Adv. Wound Care (New Rochelle).

[2] Lebeaux D., Chauhan A., Rendueles O., Beloin C. (2013). From in vitro to in vivo Models of Bacterial Biofilm-Related Infections. Pathogens.

[3] Dai T., Kharkwal G.B., Tanaka M., Huang Y.Y., Bil de Arce V.J., Hamblin M.R. (2011). Animal models of external traumatic wound infections. Virulence.

[4] Abdullahi A., Amini-Nik S., Jeschke M.G. (2014). Animal models in burn research. Cell. Mol. Life Sci..

[5] Seaton M., Hocking A., Gibran N.S. (2015). Porcine models of cutaneous wound healing. Ilar. J..

[6] Lindblad W.J. (2008). Considerations for selecting the correct animal model for dermal wound-healing studies. J. Biomater. Sci. Polym. Ed..

[7] Davidson J.M. (1998). Animal models for wound repair. Arch. Derm. Res..

[8] Gottrup F., Agren M.S., Karlsmark T. (2000). Models for use in wound healing research: A survey focusing on in vitro and in vivo adult soft tissue. Wound Repair Regen..

[9] Coolen N.A., Vlig M., van den Bogaerdt A.J., Middelkoop E., Ulrich M.M. (2008). Development of an in vitro burn wound model. Wound Repair Regen..

[10] Gurjala A.N., Geringer M.R., Seth A.K., Hong S.J., Smeltzer M.S., Galiano R.D., Leung K.P., Mustoe T.A. (2011). Development of a novel, highly quantitative in vivo model for the study of biofilm-impaired cutaneous wound healing. Wound Repair Regen..

[11] Perez R., Davis S.C. (2008). Relevance of animal models for wound healing. Wounds.

[12] Ganesh K., Sinha M., Mathew-Steiner S.S., Das A., Roy S., Sen C.K. (2015). Chronic Wound Biofilm Model. Adv. Wound Care (New Rochelle).

[13] Montagna W., Yun J.S. (1964). The Skin of the Domestic Pig. J. Investig. Derm..

[14] Bowler P.G., Duerden B.I., Armstrong D.G. (2001). Wound microbiology and associated approaches to wound management. Clin. Microbiol. Rev..

[15] Church D., Elsayed S., Reid O., Winston B., Lindsay R. (2006). Burn wound infections. Clin. Microbiol. Rev..

[16] Korber A., Schmid E.N., Buer J., Klode J., Schadendorf D., Dissemond J. (2010). Bacterial colonization of chronic leg ulcers: Current results compared with data 5 years ago in a specialized dermatology department. J. Eur. Acad. Derm. Venereol..

[17] Jeschke M.G., van Baar M.E., Choudhry M.A., Chung K.K., Gibran N.S., Logsetty S. (2020). Burn injury. Nat. Rev. Dis. Primers.

[18] Jabeen S., Clough E.C.S., Thomlinson A.M., Chadwick S.L., Ferguson M.W.J., Shah M. (2019). Partial thickness wound: Does mechanism of injury influence healing?. Burns.

[19] Zulkowski K. (2015). Wound terms and definitions. WCET J..

[20] Saleh K., Schmidtchen A. (2015). Surgical site infections in dermatologic surgery: Etiology, pathogenesis, and current preventative measures. Derm. Surg..

[21] Saleh K., Sonesson A., Persson B., Riesbeck K., Schmidtchen A. (2011). A descriptive study of bacterial load of full-thickness surgical wounds in dermatologic surgery. Derm. Surg..

[22] Wolcott R.D., Hanson J.D., Rees E.J., Koenig L.D., Phillips C.D., Wolcott R.A., Cox S.B., White J.S. (2016). Analysis of the chronic wound microbiota of 2963 patients by 16S rDNA pyrosequencing. Wound Repair Regen..

[23] Horrocks A. (2006). Prontosan wound irrigation and gel: Management of chronic wounds. Br. J. Nurs..

[24] Hirsch T., Koerber A., Jacobsen F., Dissemond J., Steinau H.U., Gatermann S., Al-Benna S., Kesting M., Seipp H.M., Steinstraesser L. (2010). Evaluation of toxic side effects of clinically used skin antiseptics in vitro. J. Surg. Res..

[25] Langtry H.D., Lamb H.M. (1998). Levofloxacin. Its use in infections of the respiratory tract, skin, soft tissues and urinary tract. Drugs.

[26] Sakamoto H., Sakamoto M., Hata Y., Kubota T., Ishibashi T. (2007). Aqueous and vitreous penetration of levofloxacin after topical and/or oral administration. Eur. J. Ophthalmol..

[27] Shepherd J., Douglas I., Rimmer S., Swanson L., MacNeil S. (2009). Development of three-dimensional tissue-engineered models of bacterial infected human skin wounds. Tissue Eng. Part C Methods.

[28] Serra R., Grande R., Butrico L., Rossi A., Settimio U.F., Caroleo B., Amato B., Gallelli L., de Franciscis S. (2015). Chronic wound infections: The role of *Pseudomonas aeruginosa* and *Staphylococcus aureus*. Expert Rev. Anti Infect..

[29] Fazli M., Bjarnsholt T., Kirketerp-Moller K., Jorgensen B., Andersen A.S., Krogfelt K.A., Givskov M., Tolker-Nielsen T. (2009). Nonrandom distribution of *Pseudomonas aeruginosa* and *Staphylococcus aureus* in chronic wounds. J. Clin. Microbiol..

[30] Sacha M., Faucon L., Hamon E., Ly I., Haltner-Ukomadu E. (2019). Ex vivo transdermal absorption of a liposome formulation of diclofenac. Biomed. Pharm..

[31] Tazrart A., Bolzinger M.A., Moureau A., Molina T., Coudert S., Angulo J.F., Briancon S., Griffiths N.M. (2017). Penetration and decontamination of americium-241 ex vivo using fresh and frozen pig skin. Chem. Biol. Interact..

[32] Donlan R.M., Costerton J.W. (2002). Biofilms: Survival mechanisms of clinically relevant microorganisms. Clin. Microbiol. Rev..

[33] Zhao G., Usui M.L., Lippman S.I., James G.A., Stewart P.S., Fleckman P., Olerud J.E. (2013). Biofilms and Inflammation in Chronic Wounds. Adv. Wound Care (New Rochelle).

[34] Wu Y.K., Cheng N.C., Cheng C.M. (2019). Biofilms in Chronic Wounds: Pathogenesis and Diagnosis. Trends Biotechnol..

[35] Tiwari V.K. (2012). Burn wound: How it differs from other wounds?. Indian J. Plast. Surg..

[36] Weaver A.J., Brandenburg K.S., Smith B.W., Leung K.P. (2019). Comparative Analysis of the Host Response in a Rat Model of Deep-Partial and Full-Thickness Burn Wounds with *Pseudomonas aeruginosa* Infection. Front. Cell. Infect. Microbiol..

[37] Pruitt B.A., McManus A.T., Kim S.H., Goodwin C.W. (1998). Burn wound infections: Current status. World J. Surg..

[38] Azeredo J., Azevedo N.F., Briandet R., Cerca N., Coenye T., Costa A.R., Desvaux M., Di Bonaventura G., Hebraud M., Jaglic Z. (2017). Critical review on biofilm methods. Crit. Rev. Microbiol..

[39] Brackman G., Coenye T. (2016). In Vitro and In Vivo Biofilm Wound Models and Their Application. Adv. Exp. Med. Biol..

[40] Wilkinson H.N., McBain A.J., Stephenson C., Hardman M.J. (2016). Comparing the Effectiveness of Polymer Debriding Devices Using a Porcine Wound Biofilm Model. Adv. Wound Care (New Rochelle).

[41] Rabin N., Zheng Y., Opoku-Temeng C., Du Y., Bonsu E., Sintim H.O. (2015). Biofilm formation mechanisms and targets for developing antibiofilm agents. Future Med. Chem..

[42] Yang S.Y., Liu Y., Mao J., Wu Y.B., Deng Y.L., Qi S.C., Zhou Y.C., Gong S.Q. (2020). The anti-biofilm and collagen-stabilizing effects of proanthocyanidin as an auxiliary endodontic irrigant. Int. Endod. J..

[43] Schmidtchen A., Pasupuleti M., Morgelin M., Davoudi M., Alenfall J., Chalupka A., Malmsten M. (2009). Boosting antimicrobial peptides by hydrophobic oligopeptide end tags. J. Biol. Chem..

[44] Myhrman E., Hakansson J., Lindgren K., Bjorn C., Sjostrand V., Mahlapuu M. (2013). The novel antimicrobial peptide PXL150 in the local treatment of skin and soft tissue infections. Appl. Microbiol. Biotechnol..

[45] McDonnell G., Haines K., Klein D., Rippon M., Walmsley R., Pretzer D. (1999). Clinical correlation of a skin antisepsis model. J. Microbiol. Methods.

[46] Phillips P.L., Yang Q., Davis S., Sampson E.M., Azeke J.I., Hamad A., Schultz G.S. (2015). Antimicrobial dressing efficacy against mature *Pseudomonas aeruginosa* biofilm on porcine skin explants. Int. Wound J..

[47] Wilkinson H.N., Iveson S., Catherall P., Hardman M.J. (2018). A Novel Silver Bioactive Glass Elicits Antimicrobial Efficacy against *Pseudomonas aeruginosa* and *Staphylococcus aureus* in an ex Vivo Skin Wound Biofilm Model. Front. Microbiol..

[48] Rizzo A.E., Beckett L.A., Baier B.S., Isseroff R.R. (2012). The linear excisional wound: An improved model for human ex vivo wound epithelialization studies. Ski. Res. Technol..

[49] Guggenheim M., Thurnheer T., Gmur R., Giovanoli P., Guggenheim B. (2011). Validation of the Zurich burn-biofilm model. Burns.

[50] Thet N.T., Alves D.R., Bean J.E., Booth S., Nzakizwanayo J., Young A.E., Jones B.V., Jenkins A.T. (2016). Prototype Development of the Intelligent Hydrogel Wound Dressing and Its Efficacy in the Detection of Model Pathogenic Wound Biofilms. ACS Appl. Mater. Interfaces.

[51] Bager C.L., Gudmann N., Willumsen N., Leeming D.J., Karsdal M.A., Bay-Jensen A.C., Hogdall E., Balslev I., He Y. (2016). Quantification of fibronectin as a method to assess ex vivo extracellular matrix remodeling. Biochem. Biophys. Res. Commun..

[52] Werthen M., Davoudi M., Sonesson A., Nitsche D.P., Morgelin M., Blom K., Schmidtchen A. (2004). *Pseudomonas aeruginosa*-induced infection and degradation of human wound fluid and skin proteins ex vivo are eradicated by a synthetic cationic polymer. J. Antimicrob. Chemother..

[53] Zeitlinger M.A., Dehghanyar P., Mayer B.X., Schenk B.S., Neckel U., Heinz G., Georgopoulos A., Muller M., Joukhadar C. (2003). Relevance of soft-tissue penetration by levofloxacin for target site bacterial killing in patients with sepsis. Antimicrob. Agents Chemother..

[54] Puthia M., Butrym M., Petrlova J., Stromdahl A.C., Andersson M.A., Kjellstrom S., Schmidtchen A. (2020). A dual-action peptide-containing hydrogel targets wound infection and inflammation. Sci. Transl. Med..

[55] Rueden C.T., Schindelin J., Hiner M.C., DeZonia B.E., Walter A.E., Arena E.T., Eliceiri K.W. (2017). ImageJ2: ImageJ for the next generation of scientific image data. BMC Bioinform..

